# Research funder required research partnerships: a qualitative inquiry

**DOI:** 10.1186/s13012-014-0176-y

**Published:** 2014-11-28

**Authors:** Shannon L Sibbald, Jacqueline Tetroe, Ian D Graham

**Affiliations:** Faculty of Health Sciences, Western University, HSB334, 1151 Richmond St, London, N6A 5B9 Canada; Department of Family Medicine and Schulich Interfaculty Program in Public Health, Schulich School of Medicine and Dentistry, Western University, London, N6G 2 M1 Canada; Canadian Institutes of Health Research (CIHR), Ottawa, K1A 0 W9 Canada; University of Ottawa, Ottawa, Canada; Ottawa Hospital Research Institute, Ottawa, Canada

**Keywords:** Grants, Partnership, Qualitative research

## Abstract

**Background:**

Researchers and funding agencies are increasingly showing interest in the application of research findings and focusing attention on engagement of knowledge-users in the research process as a means of increasing the uptake of research findings. The expectation is that research findings derived from these researcher-knowledge-user partnerships will be more readily applied when they became available. The objective of this study was to investigate the experiences, perceived barriers, successes, and opinions of researchers and knowledge-users funded under the Canadian Institutes of Health Research’s integrated Knowledge Translation funding opportunities for a better understanding of these collaborations.

**Methods:**

Participants, both researchers and knowledge-users, completed an online survey followed by an individual semi-structured phone interview supporting a mixed methods study. The interviews were analyzed qualitatively using a modified grounded theory approach.

**Results:**

Survey analysis identified three major partnership types: token, asymmetric, and egalitarian. Interview analysis revealed trends in perceived barriers and successes directly related to the partnership formation and style. While all partnerships experienced barriers, token partnerships had the most challenges and general poor perception of partnerships. The majority of respondents found that common goals and equality in partnerships did not remove barriers but increased participants’ ability to look for solutions.

**Conclusions:**

We learned of effective mechanisms and strategies used by researchers and knowledge-users for mitigating barriers when collaborating. Funders could take a larger role in helping facilitate, nurture, and sustain the partnerships to which they award grants.

## Background

Researchers and funding agencies are increasingly showing interest in the application of research findings and focusing attention on engagement of knowledge-users in the research process as a means of increasing the uptake of research findings. This general approach to research is being referred to by terms such as collaborative research [1,2], participatory action research [3-5], action-oriented research [6], community-based research [7], engaged scholarship [8,9], mode 2 knowledge production [10,11], and co-production of knowledge, despite each having its own subtle uniqueness. The Canadian Institutes of Health Research (CIHR) coined the term *integrated knowledge translation* (iKT) to refer to this concept of researchers and knowledge-users working collaboratively to address research questions [12]. The expectation of iKT research is that research findings derived from these researcher-knowledge-user partnerships will be more readily applied when they became available.

To promote this approach, funding agencies have developed iKT funding opportunities. The CIHR has three of such funding opportunities: Partnerships for Health System Improvement (PHSI), Knowledge Synthesis (KRS), and Knowledge to Action (K2A) (Table 1). Similarly, the National Health and Medical Research Council of Australia (NHMRC) has Partnership Projects and Partnership Centres, and the UK National Institute for Health Research has the Collaboration for Leadership in Applied Health Research and Care (CLAHRC) funding opportunity.

**Table 1:** **Descriptions of CIHR iKT funding opportunities at time of the study**

**CIHR iKT funding opportunity**	**Description and purpose**	**Funding**	**Definition of knowledge-user**
Partnerships for Health System Improvement (PHSI)	To support researchers and decision makers conducting applied research that will be used by health system managers and/or policymakers to strengthen Canada’s healthcare system	$600,000 total over 3 years; applicants must additionally secure a minimum of 20% of grant awarded by CIHR from other partners	Healthcare policymakers and decision makers
Knowledge Synthesis (KRS)	To support production of scoping reviews and syntheses that provide an overview of the state of knowledge on a topic; inform knowledge-users of lack/existence of evidence when making decisions; and guide researchers to primary research	$100,000 for up to 1 year (synthesis); $40,000 for up to 1 year (scoping review)	Healthcare decision makers and other researchers (will vary based on research topic)
Knowledge to Action (K2A)	To accelerate the translation of knowledge between researcher and knowledge-user in order to move knowledge into action, as well as learn about the knowledge application process	$200,000 over 2 years	Healthcare policymakers

Descriptions adapted from CIHR’s ResearchNet: http://www.cihr-irsc.gc.ca/e/47332.html#a6.2.

The CIHR undertook an evaluative inquiry on its three iKT programs with the aim of increasing its understanding of researcher-knowledge-user partnerships. The inquiry involved three steps: (1) a focused literature review, (2) a survey, and (3) qualitative interviews with a subsample of those surveyed. The focus of this paper is on the qualitative component of this inquiry (i.e., the interviews) with the purpose of addressing the gap in understanding the nature of partnerships and their impact on the process of knowledge translation. The specific questions guiding our analysis were as follows: (1) what types of experiences in partnering relationships occur with iKT grants, (2) what are the perceived barriers to partnerships, (3) what leads to successful partnerships, and (4) what is the perceived impact of partnered research?

The literature review focused on the existing literature regarding partnership research in general. Articles were found by searching using keywords, exemplar article bibliographies, reference mining, citation snowballing, and forward searching; we searched within databases and the grey literature. In total, the focused literature review identified 82 articles, 48 of which dealt specifically with researcher-knowledge-user partnerships [13]. For our purposes, we focused on researcher-knowledge-user partnerships related to the research/project team as required by the funding opportunities. The literature review identified ten dominant barriers to successful partnerships, including inadequate resources, concerns about the quality of the research, compatibility of problem solving styles among partners, level of trust between partners, turnover, power and status imbalances between researchers and knowledge-users, knowledge and skill imbalances among partners, competing agendas between researchers and knowledge-users, differences in availability and contributions, and lacking financial or personal incentives for conducting partnership research. The focused review of the literature informed the development of the survey for CIHR iKT grant recipients, as well as the qualitative interviews [14].

The survey consisted of 41 closed-ended Likert-scale questions that pertained to eight areas of inquiry; these areas included partnership details, study design, partnership outcomes, required partnerships, partnership processes, information sharing, next steps, and factors facilitating partnerships [15].

The interview guide was designed to explore respondents’ perceptions of the partnership process in general, the presence of challenges and barriers, how successful partnership research differs from non-partnered research, whether required partnerships impact the quality of research, and how CIHR can better foster this impact (interview questions available upon request).

## Methods

**Figure 1:**
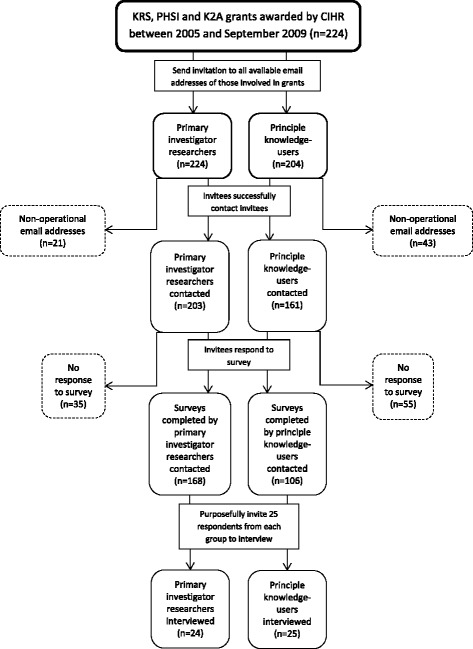
**Flow chart of researcher and knowledge-user recruitment.**

Two hundred and twenty-four CIHR iKT grants (KRS, PHSI, and K2A funding opportunities) were awarded between 2005 and September 2009 (*n* = 224 grants). Emails were sent to the 224 principal investigator researchers and 204 principal knowledge-users involved in these grants and who had provided email addresses on the application to invite them to participate in our online survey (posted in English and French). The emails were received by 203 principal investigators and 161 principal knowledge-users (the email addresses of 21 principal investigators and 43 principal knowledge-users were no longer operational; we did not attempt to track down those unable to be contacted) (91% and 79% rate of successful contact, respectively). Of those who received the emails, 173 principal investigators and 110 principal knowledge-users completed the survey (response rate of 85% and 68%, respectively). A purposeful sample of 25 researchers and 25 knowledge-users was drawn from those responding to the survey (representing about 18% of the survey respondents) based on willingness to participate in a semi-structured interview, conducted in English. Participants were contacted by a research assistant via email. Interviews were conducted over the telephone, audio-recorded, and transcribed verbatim (see Figure 1).

An iterative thematic analysis of the interview transcriptions was carried out (a modified grounded theory approach). Data analysis proceeded with two research team members reading the transcriptions once to identify and list the dominant themes expressed by the participants. Next, all research team members went through a vetting process whereby themes were assessed. To focus our analysis of the interview data, we used the participants’ responses to four questions from the survey to categorize the type of research partnership. Participants who answered negatively to all questions were categorized as token (researcher dominant). Participants who answered positively to all questions were categorized as egalitarian (researcher and knowledge-user lead). Participants who answered positive to some and negative to some questions were categorized asymmetric (researcher lead with some knowledge-user engagement) (see Table 2). Themes were first analyzed by questions and then reviewed by type of partnership. Any discrepancies were discussed and resolved; when needed, we returned to the original data to ensure accuracy and rigor of the identified themes.

**Table 2:** **Survey questions to categorize type of partnership**

**Number**	**Knowledge-user survey**		**Researcher survey**	
	**Question**	**Answer options**	**Question**	**Answer options**
8	To what extent did your partnership on this study bring your expertise as a knowledge-user into the research process?	• Not at all	To what extent did your partnership bring knowledge-user expertise into the research process?	• Not at all
		• A little		• A little
		• Somewhat		• Somewhat
		• A lot		• A lot
		• A significant amount		• A significant amount
		• Too early to tell		• Too early to tell
10	How long did it take you to develop trust in your relationship with your partner?	• Weeks	How long did it take you to develop trust in your relationship with your partner?	• Weeks
		• Months		• Months
		• Years		• Years
		• Still haven’t really done that		• Still haven’t really done that
		Please specify how long and what it took to develop trust.		Please specify how long and what it took to develop trust.
13	Who was involved in the following aspects of this study?	• Researcher(s) alone	Who was involved in the following aspects of this study?	• Researcher(s) alone
	A. Shaping the research question(s)	• Collaboration with researchers leading	A. Shaping the research question(s)	• Collaboration with researchers leading
	B. Deciding on the methodology	• Collaboration with knowledge-user(s) leading	B. Deciding on the methodology	• Collaboration with knowledge-user(s) leading
	C. Data collection and tools for development	• Collaboration with both leading equally	C. Data collection and tools for development	• Collaboration with both leading equally
	D. Interpreting the study findings and crafting messaging around them	• Knowledge-user(s) alone	D. Interpreting the study findings and crafting messaging around them	• Knowledge-user(s) alone
	E. Moving the research results into practice		E. Moving the research results into practice	
	F. Widespread dissemination and application		F. Widespread dissemination and application	
16	To what extent do you agree with this statement?	• Strongly agree	To what extent do you agree with this statement?	• Strongly agree
	“I learned a lot from my research partner while working together on this study.”	• Agree	“I learned a lot from my knowledge-user partner while working together on this study.”	• Agree
		• Neither agree nor disagree		• Neither agree nor disagree
		• Disagree		• Disagree
		• Strongly Disagree		• Strongly Disagree

The CIHR conducts evaluations under the auspices of the Treasury Board Secretariat Policy on Evaluation, and the data collected are ethically authorized under the Values and Ethics Code for the Public Service and the Privacy Act. Hence, evaluations are undertaken without Research Ethics Board review. Respondents were assured that their participation was voluntary and confidential, and decision to participate did not impact their current or future CIHR funding.

## Results

Among the 25 researchers and 25 knowledge-users purposefully drawn from the total survey respondents, we interviewed 24 researchers and 25 knowledge-users (*n* = 49; response rates of 96% and 100% respectively).Of those participants, six researchers and six knowledge-users were “matched pairs”, meaning they participated in the same grant. Thirty participants were female (61%) and 19 were male (39%). Nine (18%) of the participants had been awarded K2A grants, 24 (49%) the KRS grants, and the remaining 16 (33%) had been awarded PHSI grants. These relative numbers were proportional to the overall population of iKT grants awarded in the time period under investigation. Sixteen (33%) participants had completed their grants, and the majority (*n* = 31; 63%) did not yet finish their grants (two participants did not respond to whether they had completed their grants).

In an effort to better understand these types of required partnerships and to draw out lessons to apply to partnerships in general, we looked at partnerships as they functioned in three groups: (1) egalitarian, (2) asymmetric, and (3) token.

Egalitarian partnerships were those that were more equal and participatory in nature, and involvement in the partnership was congruent or symbiotic. Fifteen participants (six researchers and nine knowledge-users; 31%) were in an egalitarian partnership.

Twenty-seven respondents (16 researchers and 11 knowledge-users; 55%) were classified as being in an asymmetric relationship, defined as researcher lead with some knowledge-user engagement. Nearly all participants in asymmetric partnerships remained very positive about the partnership process and outcomes despite acknowledging the challenges.

Seven (three researchers and four knowledge-users; 14%) participants were part of a token partnership, or one that was very researcher dominant. Of these seven, five (71%; or 10% of the entire sample) reported the partnership as being extremely unfavorable.

We present the results in four major themes discovered through our analysis: (1) partnering process, (2) challenges to patterning, (3) facilitators of successful partnerships, and (4) impacts of partnering. Within each theme, subthemes are discussed; quotes are provided in text to provide illustrative, verbatim examples of the results. Researchers are identified by “R” and knowledge-users by “KU”; both are followed by the respondent’s unique identifier.

### Partnering process

When asked to describe the process of forming their partnership, 35% (*n* = 17) of respondents emphasized that their partnerships grew from a sharing of common interest. The majority of partnerships (over 75%) were initiated based on established relationships; seven of 49 (14%) participants had not worked with their current grant partners before. Thirty percent (*n* = 15) of respondents said that applying for funding as a formal partnership was a natural next step. Most respondents reported having a positive partnering experience; five of 49 (10%) respondents reported having a negative experience. Participants described the partnership as “enriching” and “fulfilling” (R03). Participants’ negative experiences were often a result of either a poorly defined partnership (i.e., what the partnership is, will be, or should be) or poorly defined roles within the partnership: “[This] partnership was put together really artificially, and… it just didn’t end up being an effective partnership at all” (R9). Knowledge and skill imbalances were discussed as the corollary of having diverse perspectives and experiences within a partnership: “[T]here has to be a knowledge and skill imbalance amongst team members if it’s a partnership, you each have a different skill set to bring to the table or there’s no point in having the partnership” (KU31). Both the researchers and the knowledge-users reported using their different skill sets and learning from one another to create an efficient and productive partnership:

So having this partnership and having [researchers] involved…just shapes the research differently and…I’m sure that what’s going to come out of our research will be more relevant to our knowledge-users compared to if I would have been the only one thinking about the research question. (KU47)

For participants in our study, three common factors enabled the formation of the partnership. They were as follows:

(1) An acknowledged need

[There] was this need, or wouldn’t it be great if a number of us from the four different provinces could come together to address [the same topic]…? That was something that everybody had worked on, on diverse projects over the past few years and this had come up as a potential gap. (KU16)

(2) Existing infrastructure

[T]here was already a committee in place, there was already…decision-maker buy-in to this and it was just sort of matching…the opportunity to conduct really more rigorous research on an issue that had already been identified by the organization. (R14)

(3) Appropriate timing

[W]hen this project came up…it was well-timed from our perspective because we had a variety of tools that we wanted to finish off or implement and…the community of interest, like the people who were affected by the disorders we’re trying to deal with, were starting to feel comfortable with the types of technology that we’re doing. (KU31)

Participants often reported using a “networked network” approach to finding new partners in an effort to create a stronger and more relevant partnership. For example, one researcher noted that they used “snowball sampling” (R44) to expand the partnership: “So each of us had some contact… [and] we had people on the team who we knew had certain contacts, and they brought in people”. Partnerships were dynamic in nature and often changed throughout the course of the project. The most salient example of this was using personal and professional networks to expand the partnership at various stages of the project:

[W]e’ve also engaged some newer people, so for example one new site that was involved that wasn’t involved in the beginning, joined us and… have taken a very active role in the writing up of the manuscript. (R27)

While the non-linear nature of partnerships was apparent in all partnership categories, egalitarian partnerships tended to be more open in their acceptance of this. Some asymmetric partnerships accredited their successful partnering process due to long-term or existing relationships; however, others in this category seemed to struggle through the growth phase in trying to sort out “who does what”. It was not apparent as to when a token partnership developed (early on in the grant or even pre-funding). However, it was obvious there were more barriers to overcome.

### Challenges to partnerships

When we asked participants about common barriers to partnerships, just under half of respondents encountered some form of barrier and typically identified one or two. Some respondents acknowledged barriers as part of the process:

[T]hey might not be major barriers, but they are in the mix, I think. There are issues that have to get negotiated over time. Those are…the realities, the kind of on-the-ground-realities that one faces as you’re trying to really move knowledge into practice. (R23)

Most felt that they may not surface as an actual impediment to the project or partnership: “There may be a barrier but it may not be a concern to people. They may just accept it, recognize it; they may not tie it to the outcome” (KU31). In a few cases, the barrier identified was considered detrimental to the partnerships.

The three most common barriers perceived by participants were role clarity, organizational change, and cultural differences. Budgeting concerns were discussed by some participants less frequently (for example, use of funding to attend conferences and lack of funding for research administration work).

#### Role clarity

A significant barrier identified by both researchers and knowledge-users was a lack of clearly defined expectations and roles. Researchers more so than knowledge-users saw role division as a minor barrier to having a positive partnership experience; many acknowledged “equal but different” roles. Researchers described the challenge of having to take on “the bulk of the work” (R08):

The biggest imbalance was that with decision-makers [knowledge-users] on the grant, you’re really taking the bulk of the work. Decision-makers, they tend to be there more in an expert advisory capacity. They don’t have the research skills, so I would say there is a bit of unevenness. (R05)

The majority of partnerships were driven by the researcher; knowledge-users were involved to varying extents, often contributing in more of an advisory capacity. This created a tension for some, where knowledge-users expected more involvement:

It’s appalling to me…that you’re asked to write letters of support for these researchers and sometimes you never know if the grant was funded, and you then don’t even know that it’s done…. So I think it is a power imbalance…. The researchers are in control. (KU29)

Others felt that it facilitated the partnership:

I would say [the researcher] was the initiator, did the bulk of the work and my role was more of a consultant, reactive kind of role. (KU08)

Most maintained that the role division was a minor barrier to having a positive partnership experience. Some felt that the role division reflected a power imbalance, whereas others felt that it facilitated the partnership. In some cases, respondents perceived the role division as an entrenched and serious hindrance to genuine partnerships, leading to a deeper concern of both partnership and project sustainability.

[I]t’s almost like the partners become very dependent on you, that they don’t take on the knowledge exchange or the knowledge, integration,… so we felt in a way that we were just continuing to enable them to take a bit of a back seat because we were always there to support the initiative. (R23)

#### Organizational change

Organizational change was identified as another a challenge for participants, though more so for researchers:

[O]ne of the biggest issues we faced was just so much organization change happening, it was really, really very, very difficult to work within…. We have the same players, but we had a real change in context, an organization change, and that created a lot of instability within the system itself. (R23)

Turnover was identified as a challenge and reason for needing to find new partners. While several researchers acknowledged turnover as an expected part of working with knowledge-users, it became a significant challenge in operationalizing the grant for a few:

And then a new director came in without any background in health for research and she became my boss. She was uninterested in the research,… so that was essentially severing the ties and we thought we would try to kind of rebuild them and she was an obstacle. (R06)

Turnover due to administrative changes was also cited by both researchers and knowledge-users as creating a minor challenge.

#### Cultural changes

All participants were aware of the challenge brought about by the expectations of universities to produce certain deliverables versus the potential positive impact of different deliverables on actual practice:

I can’t imagine how [competing agendas amongst team members] cannot be a barrier because academics get rewarded for different things than private or public non-research. (KU31)

The challenge became more of a barrier when it was not discussed openly and transparently:

[T]he experience, it is frustrating sometimes when… different agendas are at play…and these agendas are not lining up…. [A]gendas of the researcher [are] shaped and formed to… a great deal by the institution in which the researcher is located,…the agenda of a community-based agency and the representative of that agency…. [T]he agenda of the government partner is often times reflected by the political agenda of the day…. [T]he frustrating part is because the agenda is not clear or it is not revealed. It is not disclosed. (R03)

When alignment of research goals was not present, challenges ensued:

The problem was [the knowledge-user] vision was really different from what this process could accomplish, and it was very different from what I had anticipated and wanted out of the project. (R09)

Lack of time, or differences in how time is spent, was another prominent barrier for our participants. Time lapse between grant application submission and funding was discussed as an institutional challenge, especially given the time sensitivity of issue or solution-driven research. Knowledge-users and researchers were in agreement that time sensitivity of issue-driven research poses a challenge to how the research is done.

Differences in financial agendas also came up as a challenge for a few participants:

[My partner] tells me that his position as a [knowledge-user] is not…funded; his mandate is not research. So when he does research, there’s a cost to the organization. [T]here [are] some lack of financial incentives or personal incentives there. (R01)

Perhaps the most significant barrier identified by both researchers and knowledge-users in token partnerships was the lack of clearly defined expectations and roles, leading to frustration and negative experiences. Asymmetric partnerships also struggled with role clarity, but to a lesser extent; instead, differences in how time is spent came up as a bigger challenge. For egalitarian partnerships, organizational change and competing agendas were most often described as barriers.

### Facilitators of successful partnerships

While nearly all participants had experienced some form of barrier, the majority also discussed factors that contributed to both lessening of barriers as well as improving the likelihood of success. The four most commonly discussed factors were (1) established relationships, (2) alignment of goals/objectives, (3) skilled researchers, and (4) communication.

#### Established relationships

Seven of 49 (14%) partnerships were completely new; however, the majority of participants had some form of pre-existing partnerships. For many interview participants, having an established relationship meant that issues and barriers had been worked out prior to beginning the grant:

[We] had set up over the years all these good relationships allowing us to get access to different people through different tools and things like that, so it was kind of like it was sitting there ready. (KU31)

Participants explained that often with established relationships comes a higher degree of trust:

[I]f you have partners with whom you have worked before, you have truly created, you know, a trust-based relationship before you take on a project….You strengthen that partnership. (R07)

For some, trust was not only the foundation to a successful project, but also key in mitigating barriers: “If there’s a trusting relationship that each of the…partners knows their role, and is engaged in playing that role well, I don’t think the other barriers or challenges actually need to come about. So foundation is trust” (KU30).

#### Alignment of goals/objectives

Participants felt the partnerships were better set up to succeed when researchers’ interests were well aligned with the agendas of knowledge-users’ organizations. Knowledge-users expressed that having a shared interest facilitated the ability to take advantage of funding opportunities. Researchers saw alignment with appropriate knowledge-users as essential in ensuring successful research projects:

In terms of…figuring out which partners we wanted to be involved with and nurturing the partnership, it was pretty apparent that we had to have like somebody who was in charge of [our target population] and…involved in [our target agencies] because those are groups that are really looking to push evidence-based practice. (R39)

Alignment with the broader “research agenda” was also acknowledged (mainly by researchers) as being important:

It does require an awful lot of collaboration and…there’s a relatively small group of researchers involved in health services research in Canada,… so it becomes a bit of an incentive for people in practice to align themselves with them. (R12)

Part of alignment is also being flexible to the changes that occur. A third of respondents raised the need to recognize that both partnership and project targets must be flexible and adaptable to contextual issues. While the majority of these respondents were researchers, knowledge-users were also aware of the challenges when the project was not flexible.

#### Skilled researchers

Many participants acknowledged that it is often the more experienced researchers who apply for iKT grants; in this sense, these researchers are not only adept at partnering but also truly understand the principles of collaborative research and “whether or not you see those problems occurring” (R05). Many knowledge-users shared the belief that a skilled researcher (the “principal investigator”) with strong leadership and facilitation skills is paramount in dealing with barriers.

#### Communication

Participants acknowledged that regular, multi-modal communication was an important aspect of successful partnering. Both researchers and knowledge-users referenced various means of communication, including meetings in person or via video/web/teleconference, as well as sending project updates and summaries by email, newsletters, and blogs. There was consensus that of utmost importance is that communication is regular and that all partners are kept informed.

An entrenched division is also what contributes significantly to the “token partnerships” in which the knowledge-user plays a minimal role in the project as a whole, receives little communication from the researcher, and ultimately feels that they were asked to participate merely as a means for the researcher to acquire funding. Token partnerships did not seem to be conducive to producing successful partnerships; despite this, there were examples from both researchers and knowledge-users who acknowledged the important role that partners had in the grant. An important precondition for success in asymmetric partnerships was precise alignment between researcher interest and objectives of knowledge-users’ organizations. Egalitarian partnerships were those characterized by equal participation, as well as congruent and symbiotic involvement by both parties. Participants from egalitarian partnerships often used a “networked network” approach to identify new knowledge-users and community partners.

### Perceived impact of partnerships

Interview respondents were asked if required partnerships had more impact in comparison to grants not requiring partners. Two thirds of respondents (both researchers and knowledge-users) answered yes. The remaining third either said no or that they were uncertain. Many respondents agreed that the area of potential “impact” is where research funders need to be having a more significant role. One suggestion was to make the partners more accountable not only to the research, but also to the partnered relationship. Other participants suggested more supervision from the granting agency throughout the partnership and grant:

To ensure that the partnerships are taken seriously, it has to be part of the adjudication process, and funding ultimately has to be somehow tied to the true engagement They have to know the extent to which those researchers are going to engage their partners. (KU26)

Participants said it was sometimes difficult to measure the impact of partnership research due to the divide between impacts in the academy and those of the “real world” (KU17):

In that context a report that might actually have more impact in, in producing changes in the community or in the government than a peer-reviewed paper could ever do. But it’s not recognized in the university environment, so that can be quite frustrating. (R20)

In a few cases, participants said there had not been, nor would there be, any impact from the partnership. This most often stemmed from knowledge-users having a minimal role in the project, receiving little communication from the researcher, and ultimately feeling that they were invited to participate as simply a means for the researcher to obtain funding. Both knowledge-users and researchers considered these relationships detrimental.

Despite it being understood differently by researchers and knowledge-users, impact was commonly discussed in four ways: as (1) cultural change, (2) improved research, (3) research uptake, and (4) partnership sustainability.

#### Impact as cultural change

Many knowledge-users felt “cultural change” (KU15) regarding research in general was an important impact of the partnership. The partnership was seen as bridging the divide between academic research and the real world of policy, practice, and the community:

I think that the impact of having academic partners is that we at least begin to make sure that academics understand the realities of the practice environment, and that we don’t have this ivory tower in the academia and the real whatever in the practice setting. (KU12)

#### Impact as more relevant research

There was agreement by most researchers that having the partnership with knowledge-users makes the research impacts more relevant and “geared towards some important policy or program decision-making” (R04).

Many respondents felt that the required nature of the grant facilitated a more formal partnership, and in turn, the formalization of the partnership necessitated conditions favorable to greater impact. This included improving the broader knowledge-user perspective of researchers:

This type of grant involving partners is very, very useful, very important and it really should increase the credibility of researchers in the whole health care environment because you know, this gets us away from us being in the ivory tower. (R25)

Knowledge-users and researchers often talked about the general learning about partnering in research:

The initial plan was not necessarily carried out as expected, but it’s also the learning process that comes with such, such research proposals. (R15)

We had their input in the grant proposal. It works well because you know what they are looking for and you, you try to adapt the strategy to the kind of evidence they want to see at the end, so this is a successful approach. (R26)

#### Impact as research uptake

Participants who felt partners were engaged from the outset of the project (including proposal writing and the study design phase) also talked about knowledge-users feeling they had more ownership of the results and uptake. Both researchers and knowledge-users discussed the multiple perspectives and experiences of partnership research facilitating a broader reach of the results to multiple audiences:

So the more that people are engaged in the development of the research, the more likely they are to a) hear about the results and b) think about ways of up-taking that result because they’re invested in it.... The more you’re invested in something, the more likely you are to pay attention to it. (KU29)

#### Impact as partnership sustainability

Participants were upfront that many of the impacts of their current research might not be immediately seen; however, many knowledge-users were hopeful for continued impacts. For some participants, the egalitarian nature of the partnership was seen as being more important than the impacts of the research project. Similarly, partnership sustainability was felt to be just as important as the research outcomes:

I knew when we were applying that the partnerships would help the… program. Developing the partnership for its long-term benefits was more important than sticking to the project exactly. (R6)

While roughly half of respondents maintained that their partnerships and relationships would remain intact once projects had been completed, there were also feelings from researchers that knowledge-users needed to be able to sustain a project beyond the formal grant partnership:

[T]his kind of relates to the sustainability of the partnership and we really believe that at some point that these initiatives have to get sustained on their own; that we can’t continue to just be there you know, continuing to beat the drum, move the initiative forward; that they also have to significantly commit resources, time, etc. (R23)

For most participants, the discussion on impacts in token partnerships surrounded trying to make the partnership better from the outset and acknowledging the potential of these partnership grant opportunities. Many respondents felt that the nature of the grant facilitated a greater formality and symmetry that allowed for the partnership to have more impact on the research and results. In some cases, the partnership being egalitarian in nature was perceived to be more important than the research project impacts in and of themselves.

## Discussion

### Perceptions about partnerships

In 1979, Caplan stated that researchers and policymakers “live in separate worlds with different and often conflicting values, different rewards systems, and different languages” [16]. Despite this, researchers and policymakers (or “knowledge-users”) are now working together in an effort to improve the practical application of research, sometimes encouraged to do so by funding opportunity requirements to include a knowledge-user. It was clear through this study that researchers are aware of the importance of doing research with knowledge-user partners; for many in our study, partnership was understood as the new “way of doing research”. Even knowledge-users were aware of the current push to make research more relevant and connected to the real world: “I don’t know of any academic researchers who do not seek out partners in practice; it just is not the way of doing research in Canada any longer” (KU12). Knowing this, we sought to better understand how partnerships are actually functioning (developing, maintaining, sustaining) in this new environment. Our study supports the literature that researchers and knowledge-users share parallel views regarding the dimensions of partnerships [17], and ultimately, both feel that the benefits of partnerships outweigh the costs and barriers. Within this new environment there has been discussion around the potential for “genuine” collaboration in required partnerships. For our participants, collaborations were most often genuine. However, some felt that the forced nature of the partnerships was not conducive to true collaborative research and that it was more of a “game”, “not based on the principles of meaningful collaboration” (KU14).

Despite many of our participants using “existing” relationships to form partnerships for the grant, partnership formation was not always obvious. Our research confirms that partnerships form in a non-linear, evolving manner [18]. This non-linear nature of the partnership process was more apparent in egalitarian partnerships. Literature on partnership formulation is lacking, however [19]. Several authors have reported on “pre-cursor events/people” (such as a workshop or research liaison), as well as financial incentives (such as new grant money [20]) as impetus for or facilitators to initiate partnerships [21]. It was not clear as to when token partnerships began to form (whether early in the grant partnership or even before funding), but it was evident that further barriers needed to be surmounted.

### Perceived barriers

While barriers were experienced by all participants in this study (i.e., not unique to one type of partnership), egalitarian partners seemed to have an ability to focus on the relational and trust roles and placed a greater emphasis on overcoming the barriers as opposed to simply encountering them. Our analysis also revealed that barriers identified in the literature review were not entirely present. While cultural differences [18], time constraints, contextual issues, multiple demands [18], and staff (knowledge-user) turnover [22] seemed to be real barriers for our participants, they did not readily talk about barriers of resource constraints [23] or concerns about decreased research quality [24]. In a few instances, the notion of barriers was re-interpreted positively: Although they existed, barriers did not surface as an actual impediment to the project or partnership. We believe this finding is due to the fact that most of such partnerships developed out of established, trusting relationships. This “we-can-sort-it-out” approach was more representative of egalitarian and mature relationships. For the participants in an established relationship, issues and barriers were worked out prior to beginning the funded project. However, this finding does not imply that only new partnerships experienced problems: Despite the barriers, many newly formed partnerships were able to mitigate these in an attempt to ensure their partnerships were both meaningful and productive. Favorable outcomes (such as increased use of research) have been shown both when partners knew each other prior to formalizing their partnerships [22] and when partners did not know each other in advance [25].

### Partnership roles

Partnerships that were more successful at role clarity also seemed to be a part of long-term or existing relationships. Others seemed to struggle through the growth phase in trying to sort out “who does what”.

In many partnerships, knowledge-users took on an advisory role; for some, this was acceptable and expected. For others, however, this presented as a major challenge and a feeling they were not part of a true partnership. It is very important for partners to have a definition of both individuals’ roles in the broader partnership so that role confusion does not impede partnership success.

In particular, it is worth noting that due to the nature of the KRS grant (whereby the grant recipient conducts syntheses or scoping reviews), the researcher is often in a leadership capacity. This capacity varies however: For instance, sometimes the researcher is in charge of “researcher responsibilities” like methodology, data collection, review, and analysis, while the knowledge-user “partners are not as intensely involved” (KU03). In other instances, the researcher takes on what might be construed as an overseer role. When expectations and roles are not clearly delineated from the outset, partnerships can break down: Two of the five negative experiences reported were attributed largely to mismanaged expectations and roles.

In terms of nurturing and maintaining partnerships, this study supports existing literature that indicates the need for both consistent communication and clearly defined expectations and roles from the outset [5]. Respect for the diverse contributions of researchers and knowledge-users and accounting for the time constraints of knowledge-users were also emphasized by our respondents.

There was no consensus in our study, nor is there clear guidance in the literature to indicate if knowledge-users or researchers should be responsible for sustaining a partnership. This should be determined depending on the goals of the project or the partnership.

### Moving forward: initiation and sustainability

As researchers and knowledge-users navigate partnerships, funding agencies play a role in supporting and possibly developing partnerships. As we have seen in this study, there is no one best approach to partnerships. However, we have also shown there are a few key considerations that work to support a more successful partnership outcome: (1) a partnership built on an existing relationship, (2) alignment of researcher and knowledge-user agendas, (3) having a skilled researcher involved in the grant, and (4) regular, multi-modal communication. Granting agencies should consider these factors in adjudication, but should also consider providing guidance and support for partnerships wanting to excel in these areas.

Research funders could help foster the development of new partnerships by facilitating the interaction of researchers and knowledge-users by matching people and organizations with strong interest similarities. They might also consider taking a role in maintaining already formed partnerships by checking in and promoting egalitarian participation by both partners. More research is needed to elucidate how this might be effectively accomplished. To improve results of grant-funded partnerships, funding agencies should consider that (1) planning grants tend to enable the development of relationships and partnerships, (2) short-term grants favor opportunist partnerships, and (3) longer-term grants may encourage ongoing partnerships and sustainability of partnerships. There is also potential value in supporting ways to encourage researchers to partner with organizations, rather than solely the individuals within organizations who may move around or leave the organization.

Possibilities for supporting partnerships and facilitating their formation could include having “partnership plans” to help direct the path of the partnership and accompanying considerations to assist with overcoming barriers. Funders should consider their role in mediating partnerships and developing partnership plans.

### Study limitations

The expectation of iKT is that research findings derived from these researcher-knowledge-user partnerships will be more readily applied when they became available. At the time of our study, most grants had not been completed so respondents did not really know yet about what would be applied (or not). This also explains our focus on process and relationships because our participants could speak to that. More research is needed post-grant on research-knowledge-user partnerships to further examine this expectation.

Our study is not meant to be generalizable to all researcher-knowledge-user partnerships. The partnerships evaluated in this research were primarily positive, which most likely contributes to minimizing challenges and barriers that may have arisen (only 10% of participants had a negative experience). Our sample may be biased toward researchers and/or knowledge-users that are already in successful partnerships or those who are adept at dealing with the barriers.

## Conclusions

The goal of this study was to focus on the insights of researchers and knowledge-users who participated in funded partnership research. We found that partnerships come in many forms and the process of partnering is often an organic, non-linear endeavor. Through all of this, the challenges and barriers reported in the literature do not seem to hold up with partners who were successful in the grant competitions studied. This is perhaps due to existing and long-term relationships that may have been viewed positively by the grant reviewers and contributed to the success in the competitions. While many barriers can be mitigated and challenges overcome in researcher-knowledge-user partnerships, changes in both funding arrangements and academic performance criteria to value researcher engagement with knowledge-users could promote and empower effective partnerships with impact.

## Abbreviations

CIHR: Canadian Institutes of Health Research
iKT: Integrated knowledge translation
PHSI: Partnerships for Health System Improvement
KRS: Knowledge Synthesis
K2A: Knowledge to Action
NHMRCA: National Health and Medical Research Council of Australia
CLAHRC: Collaboration for Leadership in Applied Health Research and Care
